# Pan-keratin Immunostaining in Human Tumors: A Tissue Microarray Study of 15,940 Tumors

**DOI:** 10.1177/10668969221117243

**Published:** 2022-08-09

**Authors:** Anne Menz, Natalia Gorbokon, Florian Viehweger, Maximilian Lennartz, Claudia Hube-Magg, Lisa Hornsteiner, Martina Kluth, Cosima Völkel, Andreas M. Luebke, Christoph Fraune, Ria Uhlig, Sarah Minner, David Dum, Doris Höflmayer, Guido Sauter, Ronald Simon, Eike Burandt, Till S. Clauditz, Patrick Lebok, Frank Jacobsen, Stefan Steurer, Till Krech, Andreas H. Marx, Christian Bernreuther

**Affiliations:** 1 Institute of Pathology, 37734 University Medical Center Hamburg-Eppendorf, Hamburg, Germany; 2 Institute of Pathology, Clinical Center Osnabrueck, Osnabrueck, Germany; 3 Department of Pathology, Academic Hospital Fuerth, Fuerth Germany

**Keywords:** pan-cytokeratin, pan-keratin, tissue microarray, immunohistochemistry, cancer

## Abstract

To evaluate the efficiency of pan-keratin immunostaining, tissue microarrays of 13,501 tumor samples from 121 different tumor types and subtypes as well as 608 samples of 76 different normal tissue types were analyzed by immunohistochemistry. In normal tissues, strong pan-keratin immunostaining was seen in epithelial cells. Staining intensity was lower in hepatocytes, islets of Langerhans, and pneumocytes but markedly reduced in the adrenal cortex. Pan-keratin was positive in ≥98% of samples in 62 (83%) of 75 epithelial tumor entities, including almost all adenocarcinomas, squamous cell and urothelial carcinomas. Only 17 of 121 tumor entities (13%) had a pan-keratin positivity rate between 25% and 98%, including tumors with mixed differentiation, endocrine/neuroendocrine tumors, renal cell carcinomas, adrenocortical tumors, and particularly poorly differentiated carcinoma subtypes. The 15 entities with pan-keratin positivity in 0.9%-25% were mostly of mesenchymal origin. Reduced/absent pan-keratin immunostaining was associated with high UICC stage (p = 0.0001), high Thoenes grade (p = 0.0183), high Fuhrman grade (p = 0.0049), advanced tumor stage (p < 0.0001) and lymph node metastasis (p = 0.0114) in clear cell renal cell carcinoma, advanced pT stage (p = 0.0007) in papillary renal cell carcinoma, and with advanced stage (p = 0.0023), high grade (p = 0.0005) as well as loss of ER and PR expression (each p < 0.0001) in invasive breast carcinoma of no special type (NST). In summary, pan-keratin can consistently be detected in the vast majority of epithelial tumors, although pan-keratin can be negative a fraction of renal cell, adrenocortical and neuroendocrine neoplasms. The data also link reduced pan-keratin immunostaining to unfavorable tumor phenotype in in epithelial neoplasms.

## Introduction

Pan-keratin antibodies are mixtures of two or several antibodies that detect multiple low and high molecular weight keratins. These antibody cocktails have been designed to immunohistochemically detect all epithelial cell types irrespective of their tissues of origin with one single diagnostic tool. In surgical pathology they are typically employed to document the epithelial origin of neoplastic or non-neoplastic tissue or for detection of small metastases in lymph nodes. There are, however, limitations to the concept that pan-keratin antibodies stain all epithelial tumors and that non-epithelial tissues are “keratin negative”. For a large variety of different epithelial tumors, pan-keratin negative tumors have been described ^1–6^ and “keratin positive” mesenchymal tumors have also been reported across various mesenchymal tumor entities.^7–12^ The frequencies reported for pan-keratin negative carcinomas and pan-keratin positive non-epithelial tumors varies considerably in the literature, however. For example, pan-keratin positivity has been described in 15% to 45% of hepatocellular carcinoma carcinomas,^3,5^ 0% to 95% of adrenocortical carcinomas,^2,13,14^ 33% to 100% of clear cell^15,16^^%^ and 73% to 100% of papillary renal cell carcinomas,^17,18^ 20% to 78% of angiosarcomas,^19,20^ and 17% to 100% of leiomyosarcomas.^21,22^ These conflicting data may be caused by the use of different antibodies, immunostaining protocols, and criteria to determine “positivity” in these studies.

To generate a comprehensive dataset on the prevalence of pan-keratin positivity in epithelial and non-epithelial neoplasms, a set of tissue microarray (TMAs) was analyzed in this study that contained more than 15,500 tumor tissue samples from 121 different tumor types and subtypes as well as 76 different non-neoplastic tissue types.

## Materials and Methods

**Tissue Microarrays (TMAs).** Our normal tissue TMA was composed of 8 samples from 8 different donors for each of 76 different normal tissue types (608 samples on one slide). The tumor TMAs contained a total of 15,940 primary tumors from 121 tumor types and subtypes. The composition of normal and tumor TMAs is described in the results section. Detailed histopathological and molecular data as well as clinical-follow up data were obtained from 1157 kidney and 1475 breast cancer patients. The median follow-up time was 39 months for kidney cancer (range 1-250) and 43 months for breast cancer patients (range 1-88). All samples were retrieved from the archives of the Institutes of Pathology, University Hospital of Hamburg, Germany, the Institute of Pathology, Clinical Center Osnabrueck, Germany, and Department of Pathology, Academic Hospital Fuerth, Germany. Tissues were fixed in 4% buffered formalin and then embedded in paraffin. The TMA manufacturing process was described earlier in detail.^23,24^ In brief, one tissue spot (diameter: 0.6 mm) was transmitted from a representative tumor containing donor block into an empty recipient paraffin block. The use of archived remnants of diagnostic tissues for TMA manufacturing, their analysis for research purposes, and use of patient data were according to local laws (HmbKHG, §12) and analysis had been approved by the local ethics committee (Ethics commission Hamburg, WF-049/09). All work has been carried out in compliance with the Helsinki Declaration.

**Immunohistochemistry.** Freshly cut TMA sections were immunostained on one day and in one experiment. Slides were deparaffinized and exposed to heat-induced antigen retrieval for 5 min in an autoclave at 121 °C in a pH 9.0 buffer. The pan-cytokeratin antibody pan-keratin (recombinant rabbit, MSVA-000R, MS Validated Antibodies, GmbH, Hamburg, Germany) was applied at 37 °C for 60 min at a dilution of 1:150. Bound antibody was then visualized using the EnVision Kit (Agilent, CA, USA; #K5007) according to the manufacturer's directions. For tumor tissues, the percentage of positive neoplastic cells was estimated, and the staining intensity was semiquantitatively recorded (0, 1 + , 2 + , 3 + ). For statistical analyses, the staining results were categorized into four groups. Tumors without any staining were considered negative. Tumors with 1 + staining intensity in ≤70% of cells and 2 + intensity in ≤30% of cells were considered weakly positive. Tumors with 1 + staining intensity in >70% of cells, 2 + intensity in 31-70%, or 3 + intensity in ≤30% were considered moderately positive. Tumors with 2 + intensity in >70% or 3 + intensity in >30% of cells werde considered strongly positive.

**Statistics**. Statistical calculations were performed with JMP 14 software (SAS Institute Inc., NC, USA). Contingency tables and the chi²-test were performed to search for associations between pan-keratin and tumor phenotype. Survival curves were calculated according to Kaplan-Meier. The Log-Rank test was applied to detect significant differences between groups.

## Results

**Technical issues.** A total of 13,501 (85%) of 15,940 tumor samples were interpretable. The remaining 2439 (15%) samples were not analyzable due to the lack of unequivocal tumor cells or missing tissue spots on the TMA. For the normal tissue TMA, a sufficient number of samples was always interpretable per tissue to determine pan-keratin immunostaining.

**Figure 1.**
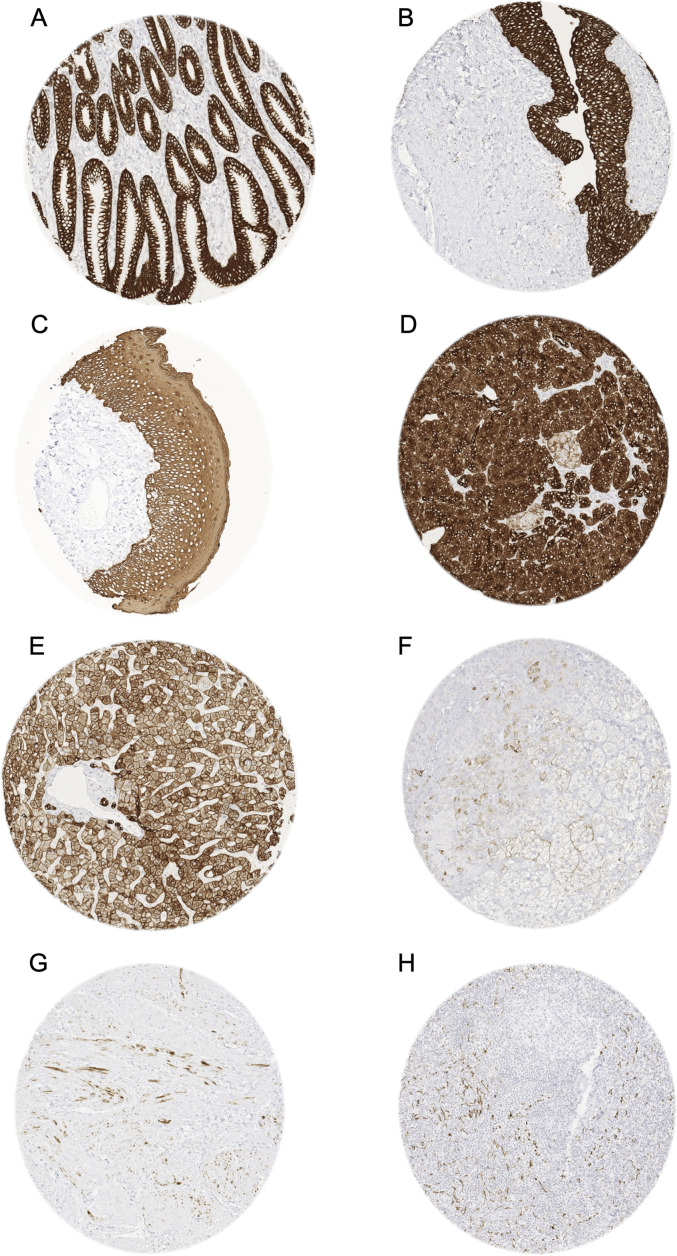
Pan-keratin immunostaining of normal tissues. The panels show a strong pan-keratin positivity of epithelial cells of the stomach (A), the urothelium of the urinary bladder (B), and the squamous epithelium of the oral cavity (C). In the pancreas, acinar cells show a strong staining while cells of islets of Langerhans stain only weakly (D). In the liver pan-keratin staining is variable (weak to moderate) in hepatocytes but strong in bile ducts (E). In the adrenal gland, only a subset of cortical cells shows a weak staining (F). In the myometrium, groups of spindle-shaped pan-keratin positive cells are found (G). In lymph nodes, a delicate fibrillar staining caused by fibroblastic reticulum cells occurs mainly in the interfollicular area (H).

**Pan-keratin in normal tissues.** A positive pan-keratin immunostaining was seen in virtually all normal epithelial and mesothelial cells. The most significant exception was the adrenal cortex, where only a fraction of cells, typically arranged in groups, fascicles or sheets, stained weakly to moderately positive. A somewhat lower, but still moderate to strong staining intensity than in most other epithelial cells was seen in hepatocytes, Langerhans islets in the pancreas, and pneumocytes of the lung. In lymph nodes, tonsil, spleen, and the thymus, a delicate fibrillar staining caused by fibroblastic reticulum cells was regularly seen, mainly in the interfollicular area. Occasional pan-keratin positive spindle shaped myofibroblasts occurred in multiple organs, especially in case of degenerative or chronic inflammatory conditions. They were, for example, seen in the media of the aorta, muscular wall of the gallbladder, placental stroma, or in the ovary in the vicinity of a corpus luteum. Groups of spindle-shaped pan-keratin positive cells were also found in the myometrium. Pan-keratin immunostaining was absent in the testis, endothelial cells, the heart, striated muscle, muscular wall of the appendix, esophagus, stomach, ileum, colon, renal pelvis and urinary bladder, corpus spongiosum of the penis, ovarian stroma, fat, testis, neurohypophysis, cerebellum and cerebrum. Representative images of pan-keratin positive normal tissues are shown in Figure 1.

**Figure 2.**
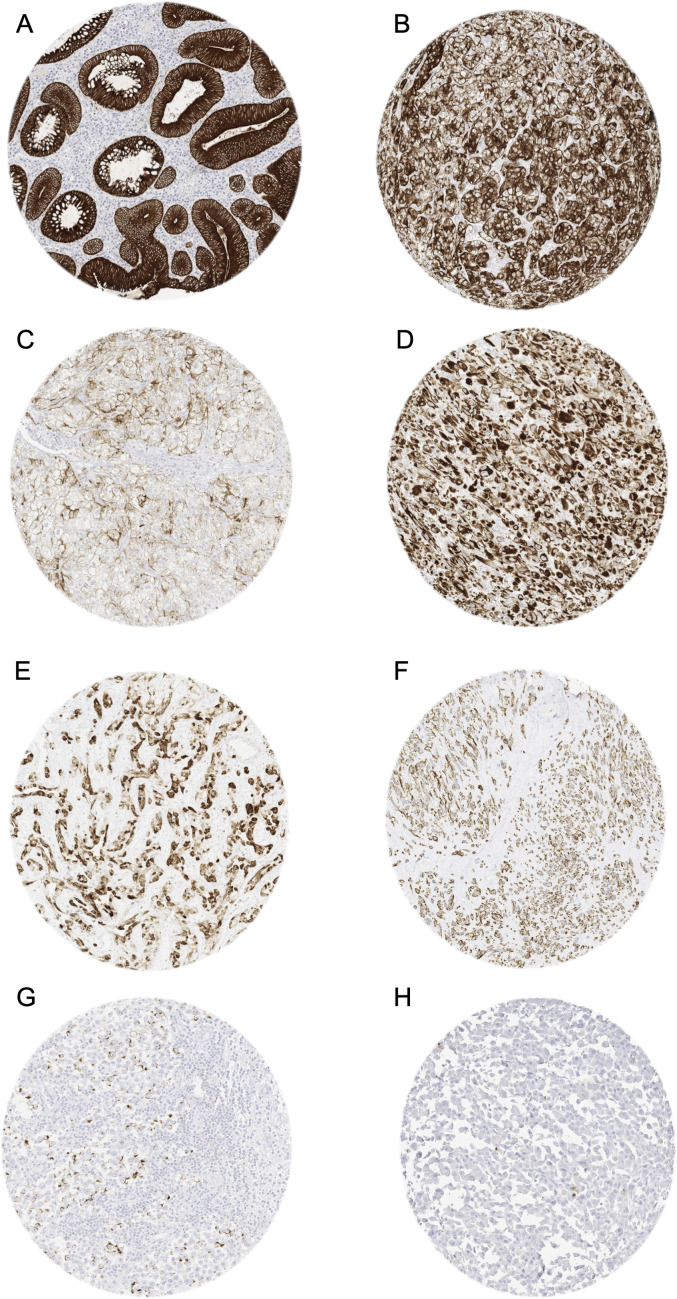
Pan-keratin immunostaining in tumors. The panels show a cytoplasmatic pan-keratin immunostaining of variable intensity in samples from a colorectal adenoma (A: strong staining), two clear cell renal cell carcinomas (B: strong; C: weak), a sarcoma NOS (D: strong), an angiosarcoma (E: strong), a gastrointestinal stromal tumor (GIST) (F: moderate), and a seminoma (G: weak). Pan-keratin immunostaining is lacking in an adrenocortical carcinoma (H).

**Figure 3.**
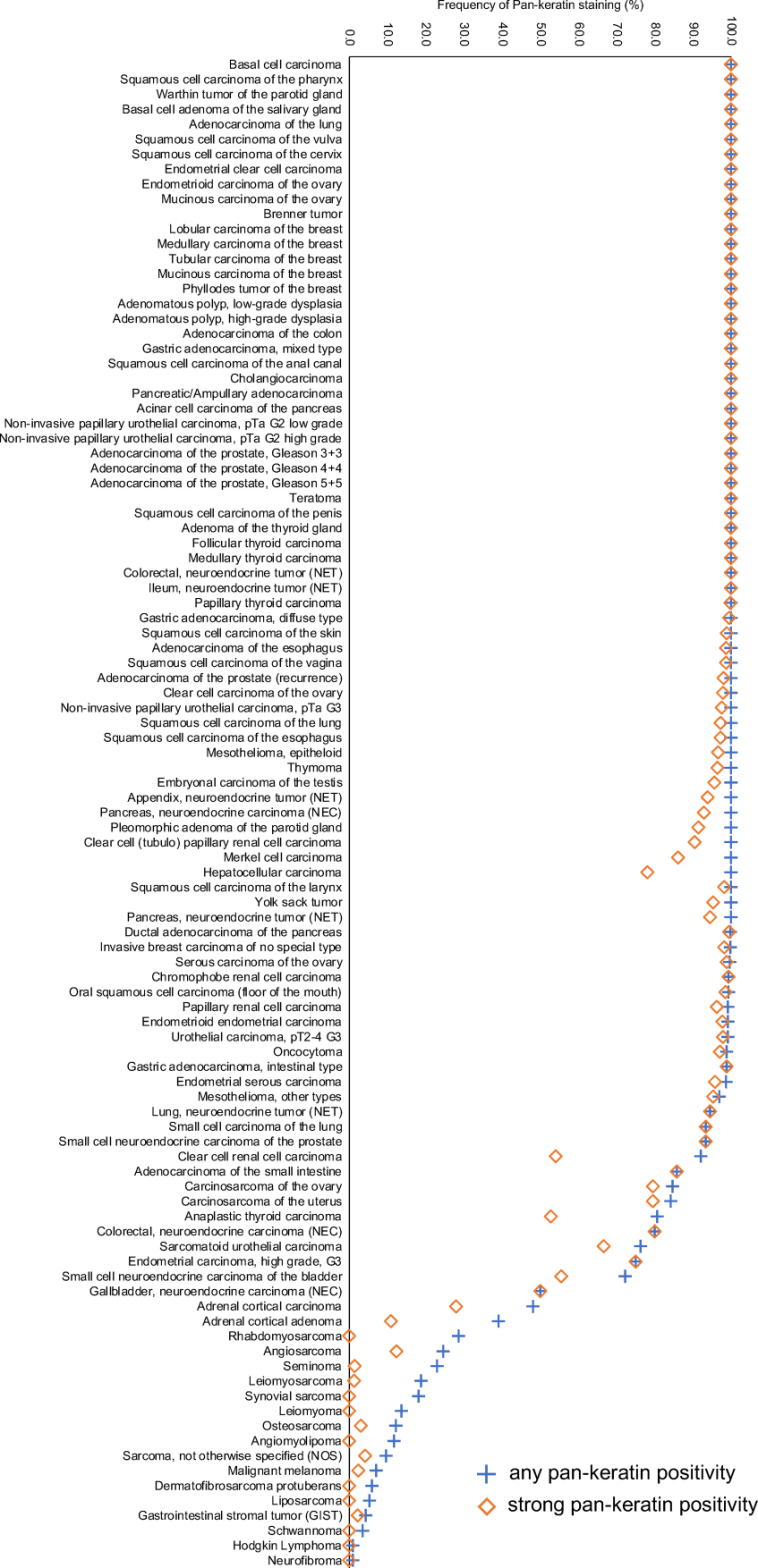
Ranking order of pan-keratin immunostaining in tumors. Both the frequency of positive tumors (blue cross) and the frequency of strongly positive tumors (orange rhombus) are shown.

**Pan-keratin in tumors.** A membranous and cytoplasmic pan-keratin immunostaining was observed in 11,323 (84%) of 13,501 analyzable tumors, including 79% with strong, 2.1% with moderate, and 2.6% with weak staining intensity. At least an occasional weak pan-keratin positivity could be detected in 101 of 121 (84%) different tumor types and tumor subtypes (Table 1). Among 75 epithelial tumor entities, the pan-keratin positivity rate was 100% in 50 (67%) and 98 – 99.9% in 12 categories (16%). These tumors included almost all adenocarcinomas, squamous cell and urothelial carcinomas. Only 17 of 121 tumor entities (13%) had a pan-keratin positivity rate between 25% and 98%. This group mainly included tumors with mixed differentiation, endocrine/neuroendocrine tumors, renal cell carcinomas, adrenocortical tumors, and particularly poorly differentiated carcinoma subtypes (anaplastic, small cell, sarcomatoid). The tumors with a pan-keratin positivity in the range of 0.9%-25% included 15 tumor entities. Most of these were of mesenchymal origin and often showed a weaker staining than epithelial neoplasms. The 20 (17%) tumor entities that were always pan-keratin negative were all of mesenchymal and hemato-lymphatic origin. Representative images of pan-keratin positive tumors are shown in Figure 2. A graphical representation of a ranking order of pan-keratin positive tumors and the observed staining intensities in these tumors is given in Figure 3. These data also show, that most tumor entities with 90%-100% positive tumors showed a strong pan-keratin immunostaining while the staining intensity decreased in tumors entities with lower positivity rates. That significant pan-keratin staining can occur in sarcomas was also confirmed by using a second independent pan-keratin antibody (Supplementary Figure 1).

**Table 1.** Pan-Keratin Immunostaining in Human Tumors.

			Pan-keratin immunostaining result				
	Tumor entity	on TMA (n)	analyzable (n)	neg. (%)	weak (%)	mod. (%)	strong (%)
**Tumors of the skin**	Basal cell carcinoma	88	82	0.0	0.0	0.0	100.0
	Benign nevus	29	25	100.0	0.0	0.0	0.0
	Squamous cell carcinoma of the skin	90	88	0.0	1.1	0.0	98.9
	Malignant melanoma	48	43	93.0	0.0	4.7	2.3
	Merkel cell carcinoma	46	43	0.0	2.3	11.6	86.0
**Tumors of the head and neck**	Squamous cell carcinoma of the larynx	110	104	0.0	0.0	1.9	98.1
	Squamous cell carcinoma of the pharynx	60	58	0.0	0.0	0.0	100.0
	Oral squamous cell carcinoma (floor of the mouth)	130	125	0.8	0.8	0.0	98.4
	Pleomorphic adenoma of the parotid gland	50	35	0.0	0.0	8.6	91.4
	Warthin tumor of the parotid gland	49	45	0.0	0.0	0.0	100.0
	Basal cell adenoma of the salivary gland	15	15	0.0	0.0	0.0	100.0
**Tumors of the lung, pleura and thymus**	Adenocarcinoma of the lung	246	176	0.0	0.0	0.0	100.0
	Squamous cell carcinoma of the lung	130	73	0.0	0.0	2.7	97.3
	Small cell carcinoma of the lung	20	15	6.7	0.0	0.0	93.3
	Mesothelioma, epitheloid	39	29	0.0	0.0	3.4	96.6
	Mesothelioma, other types	76	63	3.2	0.0	1.6	95.2
	Thymoma	29	28	0.0	0.0	3.6	96.4
**Tumors of the female genital tract**	Squamous cell carcinoma of the vagina	78	73	0.0	0.0	1.4	98.6
	Squamous cell carcinoma of the vulva	130	124	0.0	0.0	0.0	100.0
	Squamous cell carcinoma of the cervix	130	126	0.0	0.0	0.0	100.0
	Endometrioid endometrial carcinoma	236	220	0.9	0.5	0.9	97.7
	Endometrial serous carcinoma	82	70	1.4	0.0	2.9	95.7
	Carcinosarcoma of the uterus	48	44	15.9	2.3	2.3	79.5
	Endometrial carcinoma, high grade, G3	13	12	25.0	0.0	0.0	75.0
	Endometrial clear cell carcinoma	8	8	0.0	0.0	0.0	100.0
	Endometrioid carcinoma of the ovary	110	102	0.0	0.0	0.0	100.0
	Serous carcinoma of the ovary	559	509	0.4	0.2	0.6	98.8
	Mucinous carcinoma of the ovary	96	77	0.0	0.0	0.0	100.0
	Clear cell carcinoma of the ovary	50	45	0.0	0.0	2.2	97.8
	Carcinosarcoma of the ovary	47	39	15.4	5.1	0.0	79.5
	Brenner tumor	9	9	0.0	0.0	0.0	100.0
**Tumors of the breast**	Invasive breast carcinoma of no special type	1391	1152	0.3	0.6	1.0	98.1
	Lobular carcinoma of the breast	294	241	0.0	0.0	0.0	100.0
	Medullary carcinoma of the breast	26	26	0.0	0.0	0.0	100.0
	Tubular carcinoma of the breast	27	21	0.0	0.0	0.0	100.0
	Mucinous carcinoma of the breast	58	44	0.0	0.0	0.0	100.0
	Phyllodes tumor of the breast	50	43	0.0	0.0	0.0	100.0
**Tumors of the digestive system**	Adenomatous polyp, low-grade dysplasia	50	50	0.0	0.0	0.0	100.0
	Adenomatous polyp, high-grade dysplasia	50	46	0.0	0.0	0.0	100.0
	Adenocarcinoma of the colon	1932	1417	0.0	0.0	0.0	100.0
	Adenocarcinoma of the small intestine	10	7	14.3	0.0	0.0	85.7
	Gastric adenocarcinoma, diffuse type	226	158	0.0	0.0	0.6	99.4
	Gastric adenocarcinoma, intestinal type	224	162	1.2	0.0	0.0	98.8
	Gastric adenocarcinoma, mixed type	62	59	0.0	0.0	0.0	100.0
	Adenocarcinoma of the esophagus	133	77	0.0	0.0	1.3	98.7
	Squamous cell carcinoma of the esophagus	124	71	0.0	1.4	1.4	97.2
	Squamous cell carcinoma of the anal canal	91	87	0.0	0.0	0.0	100.0
	Cholangiocarcinoma	114	106	0.0	0.0	0.0	100.0
	Hepatocellular carcinoma	50	50	0.0	10.0	12.0	78.0
	Ductal adenocarcinoma of the pancreas	662	551	0.2	0.0	0.4	99.5
	Pancreatic/Ampullary adenocarcinoma	119	84	0.0	0.0	0.0	100.0
	Acinar cell carcinoma of the pancreas	15	14	0.0	0.0	0.0	100.0
	Gastrointestinal stromal tumor (GIST)	50	47	95.7	0.0	2.1	2.1
**Tumors of the urinary system**	Non-invasive papillary urothelial carcinoma, pTa G2 low grade	177	147	0.0	0.0	0.0	100.0
	Non-invasive papillary urothelial carcinoma, pTa G2 high grade	141	122	0.0	0.0	0.0	100.0
	Non-invasive papillary urothelial carcinoma, pTa G3	187	118	0.0	0.0	2.5	97.5
	Urothelial carcinoma, pT2-4 G3	1207	862	0.9	0.3	0.9	97.8
	Small cell neuroendocrine carcinoma of the bladder	18	18	27.8	16.7	0.0	55.6
	Sarcomatoid urothelial carcinoma	25	21	23.8	4.8	4.8	66.7
	Clear cell renal cell carcinoma	858	791	8.0	18.6	19.5	54.0
	Papillary renal cell carcinoma	255	236	0.8	1.3	1.7	96.2
	Clear cell (tubulo) papillary renal cell carcinoma	21	21	0.0	4.8	4.8	90.5
	Chromophobe renal cell carcinoma	131	126	0.8	0.0	0.0	99.2
	Oncocytoma	177	168	1.2	0.6	1.2	97.0
**Tumors of the male genital organs**	Adenocarcinoma of the prostate, Gleason 3 + 3	83	83	0.0	0.0	0.0	100.0
	Adenocarcinoma of the prostate, Gleason 4 + 4	80	78	0.0	0.0	0.0	100.0
	Adenocarcinoma of the prostate, Gleason 5 + 5	85	85	0.0	0.0	0.0	100.0
	Adenocarcinoma of the prostate (recurrence)	261	252	0.0	0.0	2.0	98.0
	Small cell neuroendocrine carcinoma of the prostate	17	15	6.7	0.0	0.0	93.3
	Seminoma	621	584	77.1	17.1	4.5	1.4
	Embryonal carcinoma of the testis	50	46	0.0	0.0	4.3	95.7
	Yolk sac tumor	50	42	0.0	0.0	4.8	95.2
	Teratoma	50	24	0.0	0.0	0.0	100.0
	Squamous cell carcinoma of the penis	80	78	0.0	0.0	0.0	100.0
**Tumors of endocrine organs**	Adenoma of the thyroid gland	114	106	0.0	0.0	0.0	100.0
	Papillary thyroid carcinoma	392	366	0.0	0.3	0.0	99.7
	Follicular thyroid carcinoma	158	152	0.0	0.0	0.0	100.0
	Medullary thyroid carcinoma	107	103	0.0	0.0	0.0	100.0
	Anaplastic thyroid carcinoma	45	36	19.4	13.9	13.9	52.8
	Adrenal cortical adenoma	50	46	60.9	13.0	15.2	10.9
	Adrenal cortical carcinoma	26	25	52.0	16.0	4.0	28.0
	Phaeochromocytoma	50	47	100.0	0.0	0.0	0.0
	Appendix, neuroendocrine tumor	22	16	0.0	0.0	6.3	93.8
	Colorectal, neuroendocrine tumor	11	11	0.0	0.0	0.0	100.0
	Ileum, neuroendocrine tumor	49	46	0.0	0.0	0.0	100.0
	Lung, neuroendocrine tumor	19	18	5.6	0.0	0.0	94.4
	Pancreas, neuroendocrine tumor	98	91	0.0	1.1	4.4	94.5
	Colorectal, neuroendocrine carcinoma	12	10	20.0	0.0	0.0	80.0
	Gallbladder, neuroendocrine carcinoma	4	4	50.0	0.0	0.0	50.0
	Pancreas, neuroendocrine carcinoma	14	14	0.0	7.1	0.0	92.9
**Tumors of haemotopoetic and lymphoid tissues**	Hodgkin Lymphoma	103	103	99.0	1.0	0.0	0.0
	Non-Hodgkin Lymphoma	62	61	100.0	0.0	0.0	0.0
	Small lymphocytic lymphoma, B-cell type	50	50	100.0	0.0	0.0	0.0
	Diffuse large B cell lymphoma	114	114	100.0	0.0	0.0	0.0
	Follicular lymphoma	88	88	100.0	0.0	0.0	0.0
	T-cell Non Hodgkin lymphoma	24	24	100.0	0.0	0.0	0.0
	Mantle cell lymphoma	18	18	100.0	0.0	0.0	0.0
	Marginal zone lymphoma	16	16	100.0	0.0	0.0	0.0
	Diffuse large B-cell lymphoma in the testis	16	16	100.0	0.0	0.0	0.0
	Burkitt lymphoma	5	3	100.0	0.0	0.0	0.0
**Tumors of soft tissue and bone**	Tenosynovial giant cell tumor	45	45	100.0	0.0	0.0	0.0
	Granular cell tumor	53	46	100.0	0.0	0.0	0.0
	Leiomyoma	50	44	86.4	13.6	0.0	0.0
	Leiomyosarcoma	87	85	81.2	12.9	4.7	1.2
	Liposarcoma	132	116	94.8	3.4	1.7	0.0
	Malignant peripheral nerve sheath tumor	13	13	100.0	0.0	0.0	0.0
	Myofibrosarcoma	26	26	100.0	0.0	0.0	0.0
	Angiosarcoma	73	65	75.4	9.2	3.1	12.3
	Angiomyolipoma	91	86	88.4	10.5	1.2	0.0
	Dermatofibrosarcoma protuberans	21	17	94.1	5.9	0.0	0.0
	Ganglioneuroma	14	12	100.0	0.0	0.0	0.0
	Kaposi sarcoma	8	5	100.0	0.0	0.0	0.0
	Neurofibroma	117	107	99.1	0.9	0.0	0.0
	Sarcoma, not otherwise specified (NOS)	75	73	90.4	4.1	1.4	4.1
	Paraganglioma	41	41	100.0	0.0	0.0	0.0
	Ewing Sarcoma	23	15	100.0	0.0	0.0	0.0
	Rhabdomyosarcoma	7	7	71.4	28.6	0.0	0.0
	Schwannoma	121	117	96.6	3.4	0.0	0.0
	Synovial sarcoma	12	11	81.8	18.2	0.0	0.0
	Osteosarcoma	43	33	87.9	0.0	9.1	3.0
	Chondrosarcoma	38	18	100.0	0.0	0.0	0.0

**Figure 4.**
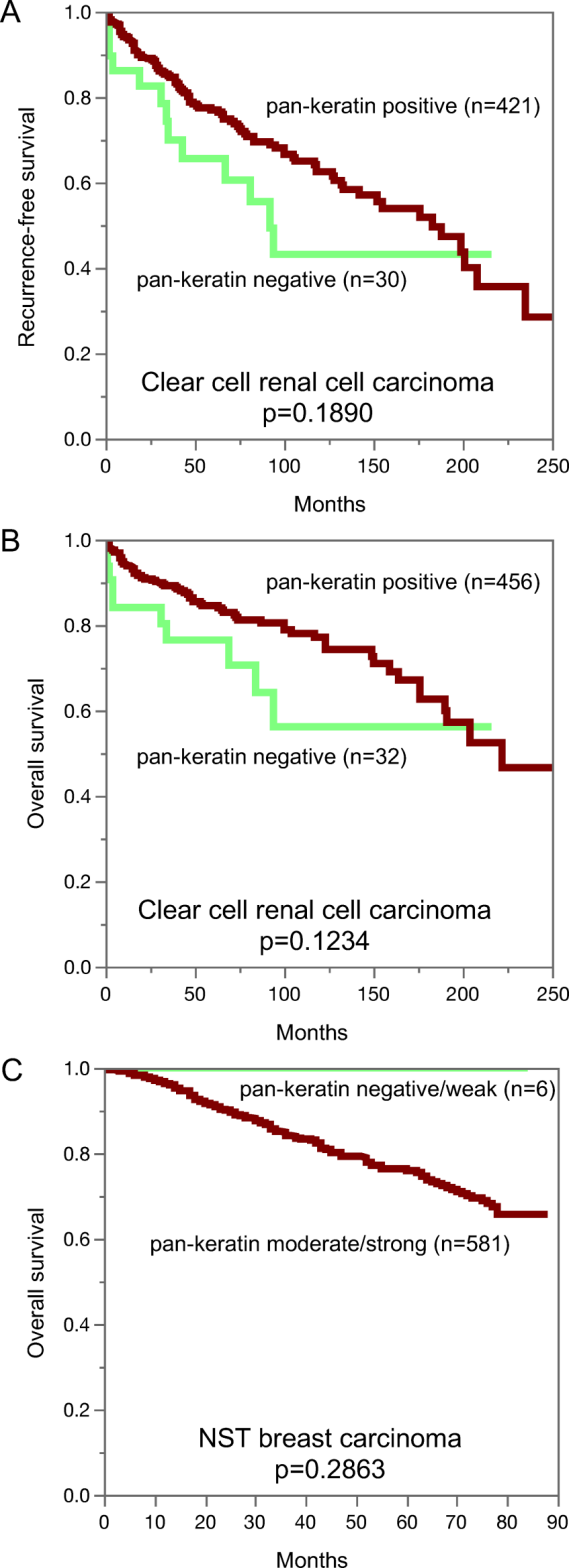
Pan-keratin immunostaining and recurrence-free survival (A) and overall survival (B) in clear cell renal cancer. Pan-keratin immunostaining in NST breast cancer and overall survival (C).

**Pan-keratin immunostaining, tumor phenotype, and prognosis.** The relationship between pan-keratin immunostaining and clinico-pathological data could be analyzed in breast and kidney cancer (Table 2). Reduced or absent pan-keratin immunostaining was associated with high UICC stage (p = 0.001), high Thoenes grade (p = 0.0183), high Fuhrman grade (p = 0.0049), advanced tumor stage (p < 0.0001) as well as lymph node metastasis (p = 0.0114) in clear cell renal cell carcinomas, with high UICC stage (p = 0.0062) and advanced pT stage (p = 0.0007) in papillary renal cell carcinoma, and with advanced stage (p = 0.0023), high grade (p = 0.0005) as well as loss of estrogen receptor (ER) and progesteron receptor (PR) expression and a triple-negative status (p < 0.0001 each) in invasive breast carcinoma of no special type (NST). Despite a tendency towards shortened recurrence free (Figure 4a; p = 0.1890) and overall survival (Figure 4b, p = 0.1234) for pan-keratin negative clear cell renal cell carcinomas, this difference did not reach statistical significance. Reduced pan-keratin immunostaining was not statistically linked to poor outcome in NST (Figure 4c, p = 0.2863) but there were only 6 patients with reduced pan-keratin staining for which clinical follow-up data were available.

**Table 2.** Pan-Keratin Immunostaining and Tumor Phenotype in Breast and Clear Cell Renal and Papillary Renal Cell Cancer.

				Pan-keratin IHC result				
			n	negative (%)	weak (%)	moderate (%)	strong (%)	*P*
**Breast carcinoma of no special type**	Primary Tumor	pT1	547	0.5	0.5	0.0	98.9	0.0023
		pT2	400	0.0	1.0	1.5	97.5	
		pT3-4	89	0.0	0.0	3.4	96.6	
	Grade	G1	171	0.6	0.6	0.0	98.8	0.0005
		G2	538	0.2	0.4	0.0	99.4	
		G3	366	0.3	1.1	2.7	95.9	
	Regional Lymph Nodes	pN0	444	0.0	0.7	0.9	98.4	0.7401
		pN1	217	0.0	0.5	0.9	98.6	
		pN2	72	0.0	0.0	0.0	100.0	
		pN3	50	0.0	0.0	0.0	100.0	
	HER2 status	negative	840	0.4	0.6	1.1	98.0	0.5544
		positive	114	0.0	0.0	0.9	99.1	
	ER status	negative	198	0.5	1.5	4.5	93.4	<0.0001
		positive	713	0.1	0.3	0.1	99.4	
	PR status	negative	381	0.3	0.8	2.6	96.3	<0.0001
		positive	564	0.2	0.4	0.0	99.5	
	Triple negative	no	749	0.1	0.3	0.3	99.3	<0.0001
		yes	133	0.8	2.3	6.0	91.0	
							
**Clear cell renal cell carcinoma**								
	ISUP grade	1	236	7.6	17.8	21.2	53.4	0.1907
		2	248	7.7	17.3	21.0	54.0	
		3	206	7.8	20.9	19.4	51.9	
		4	42	21.4	26.2	9.5	42.9	
	Fuhrmann grade	1	38	7.9	5.3	15.8	71.1	0.0049
		2	443	7.4	18.1	21.2	53.3	
		3	210	7.6	21.0	21.0	50.5	
		4	50	20.0	26.0	6.0	48.0	
	Thoenes grade	1	268	6.3	17.2	20.1	56.3	0.0183
		2	406	8.6	18.0	21.4	52.0	
		3	67	14.9	29.9	9.0	46.3	
	UICC stage	1	340	3.5	15.0	17.9	63.5	0.001
		2	35	8.6	8.6	22.9	60.0	
		3	91	14.3	24.2	17.6	44.0	
		4	74	13.5	14.9	21.6	50.0	
	Primary Tumor	1	446	4.9	17.0	17.9	60.1	<0.0001
		2	76	10.5	19.7	26.3	43.4	
		3-4	214	15.0	22.4	21.0	41.6	
	Regional Lymph Nodes	0	127	9.4	22.0	22.0	46.5	0.0114
		≥1	18	16.7	0.0	11.1	72.2	
	Distant Metastasis	0	113	6.2	15.9	22.1	55.8	0.192
		≥1	76	15.8	14.5	22.4	47.4	
							
**Papillary renal cell carcinoma**								
	ISUP grade	1	40	0.0	0.0	2.5	97.5	0.933
		2	92	1.1	2.2	1.1	95.7	
		3	58	1.7	1.7	3.4	93.1	
		4	1	0.0	0.0	0.0	100.0	
	Fuhrmann grade	1	2	0.0	0.0	0.0	100.0	0.9965
		2	130	0.8	1.5	1.5	96.2	
		3	56	1.8	1.8	3.6	92.9	
		4	3	0.0	0.0	0.0	100.0	
	Thoenes grade	1	49	0.0	0.0	2.0	98.0	0.664
		2	133	1.5	2.3	2.3	94.0	
		3	9	0.0	0.0	0.0	100.0	
	UICC stage	1	104	0.0	1.0	0.0	99.0	0.0062
		2	18	0.0	0.0	5.6	94.4	
		3	5	20.0	0.0	40.0	40.0	
		4	12	0.0	0.0	0.0	100.0	
	Primary Tumor	1	133	0.0	0.8	0.0	99.2	0.0007
		2	37	2.7	2.7	2.7	91.9	
		3-4	15	6.7	6.7	20.0	66.7	
	Regional Lymph Nodes	0	19	0.0	0.0	5.3	94.7	0.423
		≥1	7	0.0	0.0	0.0	100.0	
	Distant Metastasis	0	27	0.0	0.0	3.7	96.3	0.4935
		≥1	7	0.0	0.0	0.0	100.0	
							

## Discussion

The standardized analysis of 13,501 tumors provided a comprehensive overview on pan-keratin immunostaining in different tumor types. That the graphical representation of the frequencies of pan-keratin immunostaining among 121 different tumor entities resulted in an S-shaped curve reflects the fact that intense pan-keratin immunostaining is common in epithelial neoplasms while non-epithelial tumors are usually pan-keratin negative.

Among epithelial tumor entities, 50 of 75 (67%) showed Pan-keratin positivity in 100% of tumors and 12 (16%) were positive in ≥98% of tumors. These entities include virtually all important types of adenocarcinomas and squamous cell carcinomas. We assume that a fraction of the few negative tumors in these cancer types may be caused by technical issues. Some unexpected negative immunostaining results always occur in TMAs because not all tissues are properly fixed in all areas.^25^ Unequal immunostaining in tissues can results in an immunostaining gradient across a tissue block and can thus cause false negative immunostaining, if TMA cores are taken from areas with poor reactivity.^26^ It is likely that – in at least a fraction of these tumors - some immunoreactive areas will be found if larger tissue samples, perhaps derived from different blocks, are analyzed.

The group of tumor entities with a pan-keratin positivity in 25% to 98% of analyzed tumors made up for only 17 (13%) of analyzed entities. Most of these tumors could be categorized into the following 5 groups: tumors with mixed differentiation, endocrine/neuroendocrine tumors, kidney tumors, adrenocortical tumors, and particularly poorly differentiated carcinoma subtypes. The group of tumors with mixed epithelial-mesenchymal differentiation includes carcinosarcoma of the uterus and the ovary, phyllodes tumor of the breast, teratoma of the testis, and malignant mesothelioma. In these tumors, epithelial but not mesenchymal tumor areas are pan-keratin positive. The pan-keratin TMA result therefore depends on whether epithelial components are present in the TMA spot or not. The rather low positivity rate in kidney cancers reflects the fact that these tumors have low cytokeratin levels and tend to express vimentin instead.^27,28^

For adrenocortical, neuroendocrine and endocrine tumors, the intermediate positivity rate appears to mirror the rather low pan-keratin immunostaining in corresponding normal cells. Hepatocellular carcinoma, another tumor for which reduced cytokeratin has often been reported ^5,29,30^ was always pan-keratin positive in this study, although staining was only weak or moderate in 22% of tumors.

Very poorly differentiated cancers, such as small cell and sarcomatoid cancers as well as anaplastic cancers of the thyroid, often showed lower pan-keratin immunostaining rates as compared to corresponding normal tissues and more differentiated tumors from these organs. This may reflect that a reduced expressions of keratins is a feature of tumors dedifferentiation that can occur during cancer progression. This interpretation is also in line with our findings in kidney and breast carcinomas showing significant associations between reduced pan-keratin immunostaining and several unfavorable phenotypic tumor features. Other investigators had previously also described significant correlations between reduced expression of specific keratins ^31–34^ or reduced pan-keratin immunostaining ^35^ and poor patient prognosis or unfavorable tumor phenotype in various tumor types. A reduced expression of keratins in tumors derived from keratin positive progenitor cells is likely to represent a feature of cellular dedifferentiation which regularly goes along with cancer progression. Our data also emphasize that pan-keratin immunostaining is not uncommon in mesenchymal tumors although the expression levels are usually lower in these neoplasms as compared to carcinomas. In this study, pan-keratin immunostaining was observed in 13 mesenchymal tumor entities with highest rates in rhabdomyosarcomas (29%) and angiosarcomas (25%). These findings are in line with earlier studies describing keratin expression in 20%, 36%, 100% of rhabdomyosarcomas ^36–38^ and in 20%, 33%, and 88% of angiosarcomas.^19,20,39^ Given the significant pan-keratin staining in the majority of cells is several sarcomas of different types, cytokeratin positivity – even if strong – should not automatically lead to a diagnosis of “sarcomatoid carcinoma”.

In summary, our data show that pan-keratin can consistently be detected in the vast majority of epithelial tumors but also identify renal cell, hepatocellular, adrenocortical and neuroendocrine neoplasms as tumors lacking pan-keratin immunostaining in a fraction of tumors. Moreover, pan-keratin immunostaining - usually at lower levels - can also occur in a broad range of mesenchymal tumors.

## Supplemental Material

sj-pdf-1-ijs-10.1177_10668969221117243 - Supplemental material for Pan-keratin Immunostaining in Human Tumors: A Tissue Microarray Study of 15,940 Tumors Click here for additional data file. Supplemental material, sj-pdf-1-ijs-10.1177_10668969221117243 for Pan-keratin Immunostaining in Human Tumors: A Tissue Microarray Study of 15,940 Tumors by Anne Menz, Natalia Gorbokon, Florian Viehweger, Maximilian Lennartz, Claudia Hube-Magg, Lisa Hornsteiner, Martina Kluth, Cosima Völkel, Andreas M. Luebke, Christoph Fraune, Ria Uhlig, Sarah Minner, David Dum, Doris Höflmayer, Guido Sauter, Ronald Simon, Eike Burandt, Till S. Clauditz, Patrick Lebok, Frank Jacobsen, Stefan Steurer, Till Krech, Andreas H. Marx and Christian Bernreuther in International Journal of Surgical Pathology

sj-docx-2-ijs-10.1177_10668969221117243 - Supplemental material for Pan-keratin Immunostaining in Human Tumors: A Tissue Microarray Study of 15,940 Tumors Click here for additional data file. Supplemental material, sj-docx-2-ijs-10.1177_10668969221117243 for Pan-keratin Immunostaining in Human Tumors: A Tissue Microarray Study of 15,940 Tumors by Anne Menz, Natalia Gorbokon, Florian Viehweger, Maximilian Lennartz, Claudia Hube-Magg, Lisa Hornsteiner, Martina Kluth, Cosima Völkel, Andreas M. Luebke, Christoph Fraune, Ria Uhlig, Sarah Minner, David Dum, Doris Höflmayer, Guido Sauter, Ronald Simon, Eike Burandt, Till S. Clauditz, Patrick Lebok, Frank Jacobsen, Stefan Steurer, Till Krech, Andreas H. Marx and Christian Bernreuther in International Journal of Surgical Pathology
