# Supplementary figures and images for: Pan-keratin Immunostaining in Human Tumors: A Tissue Microarray Study of 15,940 Tumors
Source: Int J Surg Pathol. 2022 Aug 9;31(6):927–38. doi: 10.1177/10668969221117243 (PMC10492441; doi:10.1177/10668969221117243)

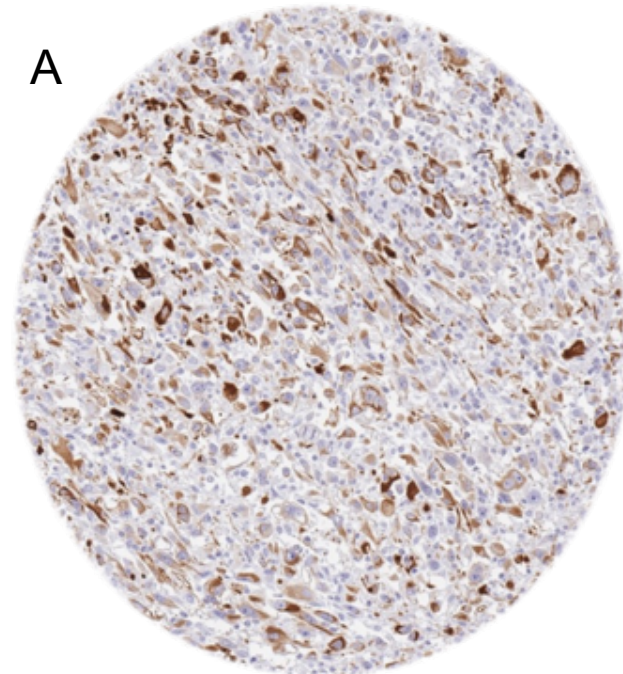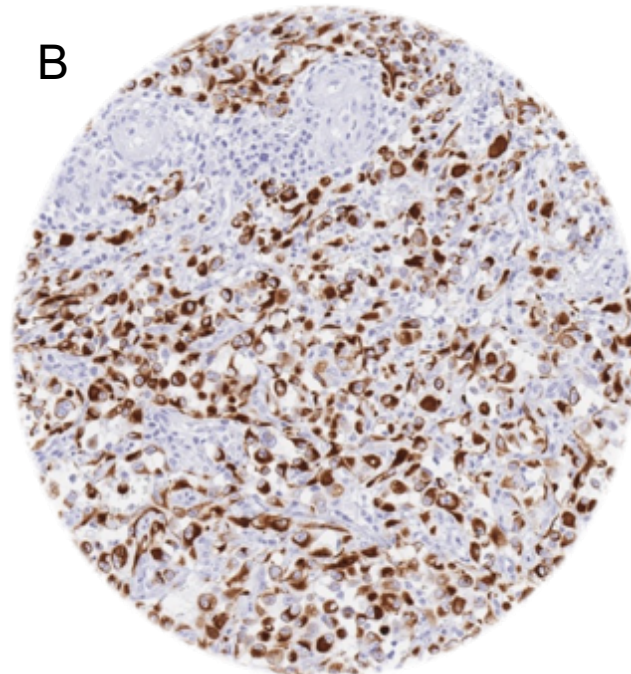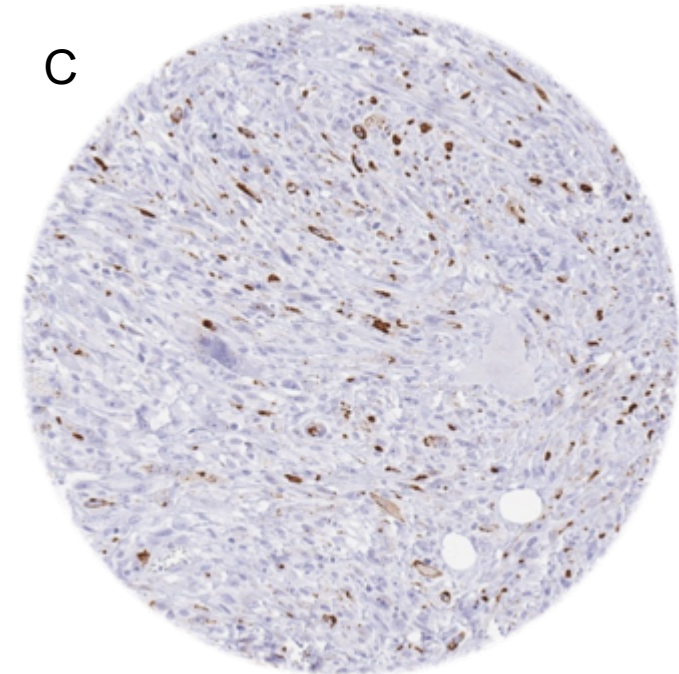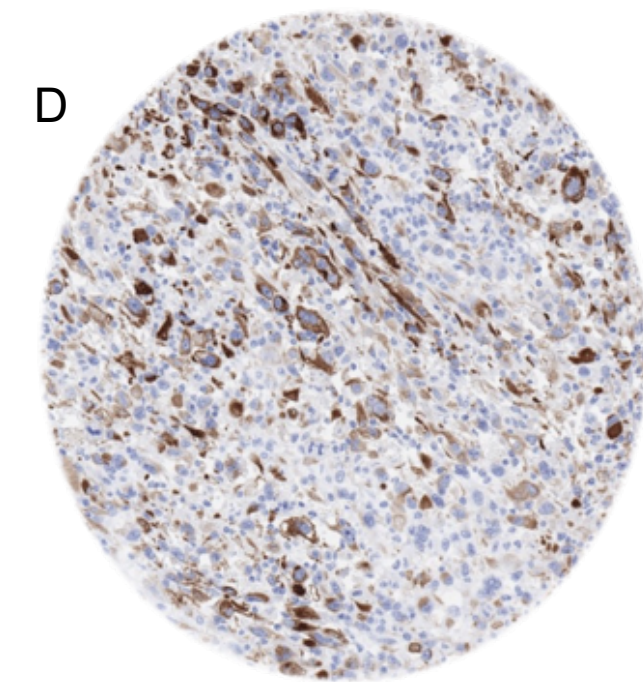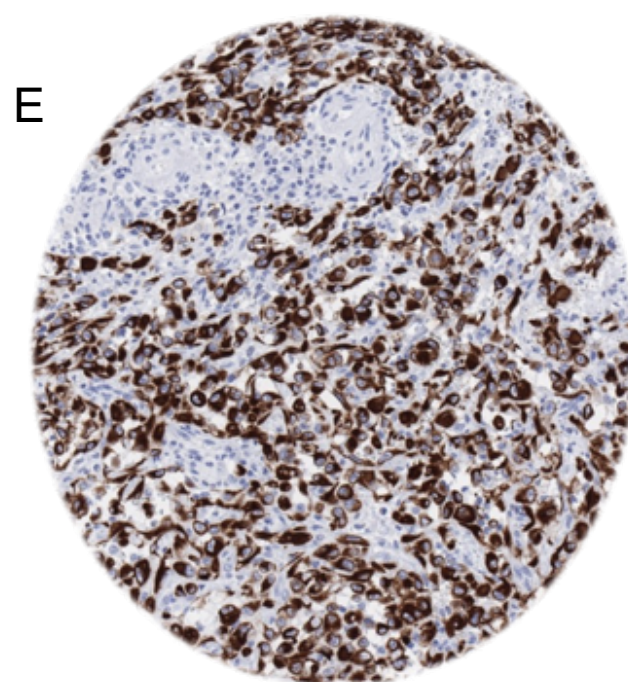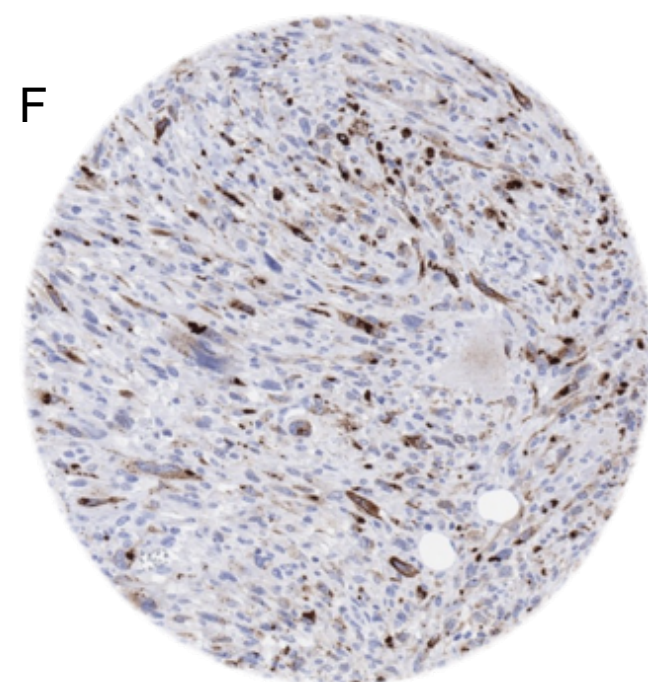

Supplement: sj-pdf-1-ijs-10.1177_10668969221117243 - Supplemental material for Pan-keratin Immunostaining in Human Tumors: A Tissue Microarray Study of 15,940 Tumors [file sj-pdf-1-ijs-10.1177_10668969221117243.pdf]
