# Supplementary material for: Pan-keratin Immunostaining in Human Tumors: A Tissue Microarray Study of 15,940 Tumors
Source: Int J Surg Pathol. 2022 Aug 9;31(6):927–38. doi: 10.1177/10668969221117243 (PMC10492441; doi:10.1177/10668969221117243)
Supplement: sj-docx-2-ijs-10.1177_10668969221117243 - Supplemental material for Pan-keratin Immunostaining in Human Tumors: A Tissue Microarray Study of 15,940 Tumors [file sj-docx-2-ijs-10.1177_10668969221117243.docx]

**Supplementary Figure 1:** **IHC validation by comparison of antibodies.** The panels show a full concordance of immunostaining results obtained by two independent pan-keratin antibodies in sarcomas. Using the CKpan antibody MSVA-000R, an unequivocal cytoplasmic immunostaining was seen in individual sarcoma NOS (A), angiosarcoma (B), and liposarcoms cases (C). Using the panepithelial antibody AE1/3, a similar staining pattern was observed in these sarcoma NOS (D), angiosarcoma (E), and liposarcoma samples (F). The images A-C and D-F are from consecutive tissue sections.
